# Fermented milk containing *Lactobacillus paracasei* and *Glycyrrhiza glabra* has a beneficial effect in patients with *Helicobacter pylori* infection: A randomized, double-blind, placebo-controlled study

**DOI:** 10.1097/MD.0000000000016601

**Published:** 2019-08-30

**Authors:** Jin Young Yoon, Jae Myung Cha, Seong Soo Hong, Hyung Kyung Kim, Min Seob Kwak, Jung Won Jeon, Hyun Phil Shin

**Affiliations:** a Department of Internal Medicine, Division of Gastroenterology, Kyung Hee University Hospital at Gang Dong, Kyung Hee University School of Medicine; b Division of Gastroenterology, Vievis Namuh Hospital; c Department of Medicine, Graduate School, Kyung Hee University, Seoul, Korea.

**Keywords:** fermented milk, *Glycyrrhiza glabra*, *Helicobacter pylori*, *Lactobacillus paracasei*, probiotics

## Abstract

**Background:**

*Lactobacillus paracasei* and *Glycyrrhiza glabra* have been reported as having beneficial effects on *Helicobacter pylori* infection. We aimed to assess the efficacy and safety of fermented milk containing *L paracasei* HP7 and *G glabra* in patients with *H pylori* infection.

**Methods:**

This multicenter, prospective, randomized, double-blind, placebo-controlled clinical trial was conducted in 2 hospitals from April to December 2017. Patients with *H pylori* infection were randomized into either the treatment group (fermented milk with *L paracasei* HP7 and *G glabra*) or placebo group (fermented milk only) once daily for 8 weeks. The primary endpoint was the gastric load of *H pylori* measured by ^13^C-urea breath test (UBT). Secondary endpoints were histologic and clinical improvement.

**Results:**

A total of 142 patients were randomly allocated to the treatment (n = 71) or placebo groups (n = 71). Compared to baseline data, the quantitative value of ^13^C-UBT at 8 weeks was significantly reduced in the treatment group (from 20.8 ± 13.2% to 16.9 ± 10.8%, *P* = .035), but not in the placebo group (*P* = .130). Chronic inflammation improved significantly only in the treatment group (*P* = .013), whereas the neutrophil activity deteriorated significantly only in the placebo group (*P* = .003). Moreover, the treatment group had significant improvement in gastrointestinal symptoms (*P* = .049) and quality of life (*P* = .029). No serious adverse events were observed.

**Conclusion:**

The combination of fermented milk containing *L paracasei* and *G glabra* reduced *H pylori* density and improved histologic inflammation. However, their mechanisms of action should be elucidated in further studies.

## 1 Introduction

*Helicobacter pylori* is a gram-negative spiral-shaped microaerophilic bacteria colonizing the human gastric epithelial cell with more than 50% prevalence and is associated with gastric malignancy as well as chronic atrophic gastritis and peptic ulcer.^[1–3]^ The 1st-line standard regimen used for the eradication of *H pylori* infection is composed of 2 antibiotics and 1 proton-pump inhibitor (PPI).^[4]^ Unfortunately, this triple therapy has limited efficacy because of antibiotic resistance of the pathogen and poor compliance due to adverse effects.^[5]^ A large number of studies have been conducted to identify the optimal regimen for *H pylori* eradication; however, its success rate still remains challenging. Even though several studies have evaluated the effects of supplementation with probiotics or alternatives along with standard triple regimen in *H pylori* eradication,^[6,7]^ no studies showed a beneficial effect in patients with *H pylori* infection.

Gut microflora may be involved in the pathophysiology of various diseases through immunoregulatory function.^[8,9]^ Dysbiosis, that is, imbalance between the protective and harmful gut microbiome, could lead to stimulation of inflammatory response, dysfunction of the intestinal epithelium, and increased permeability of the mucosa. Therefore, probiotic supplementation might be useful for the management of inflammatory conditions. Recently, probiotics have been added in the treatment of *H pylori* infection, as it might reduce the adverse effects of antibiotics and improve the success rate of *H pylori* eradication.^[8,10,11]^ Of these probiotic strains, the genera Lactobacillus has been the most frequently investigated for the anti-*H pylori* activity in animal studies.^[12,13]^ The genus *Lactobacillus* can produce extracellular polysaccharides (EPSs),^[14]^ which modulate the host's immune response by either stimulating or suppressing the response in inflammatory disorders.^[15]^*Lactobacillus paracasei*, which is one of the representative *Lactobacillus* strains producing large amounts of EPS,^[16]^ has demonstrated positive effects on health and disease in many clinical trials,^[17–26]^ such as improvement of symptoms in gastroenteritis,^[18–20]^ colonic diverticulitis,^[21,22]^ or irritable bowel syndrome^[23]^ and lowering of serum triacylglycerol level.^[24,25]^ Currently, *L paracasei* has been widely used as a single probiotic or in combination with other prebiotics.^[26]^

*Glycyrrhiza glabra* (licorice) has been traditionally used as a herbal medicine in various countries for many years.^[27,28]^*G glabra* has been reported for various clinical effects, such as antiinflammatory, antimicrobial, antiviral, antiprotozoal, antioxidative, hepatoprotective, anxiolytic, and even antitumor.^[29]^ Furthermore, a root extract of *G glabra* is also reported to have favorable gastrointestinal effects, such as antiulcer activity, gastric epithelial cell protection, and gastrointestinal motility regulation.^[30–32]^ In vitro, aqueous extract of *G glabra* suppressed *H pylori* activity through inhibiting the adhesion of *H pylori* to the gastric cells.^[33,34]^

In this study, we aimed to assess the efficacy and safety of fermented milk containing *L paracasei* HP7 and *G glabra* in patients with *H pylori* infection with a randomized, double-blind, placebo-controlled trial.

## 2 Materials and methods

### 2.1 Study population

Patients between the ages of 19 and 70 years were eligible, if they were confirmed with *H pylori* infection by ^13^C-urea breath test (UBT), *Campylobacter*-like organism (CLO) test, or histologic examination (Giemsa or hematoxylin and eosin stain) within 1 year. Patients were excluded if they had used nonsteroidal antiinflammatory drugs, corticosteroids, antimicrobials, acid-suppressing medications (such as PPIs and H2-blocker), bismuth compounds, or probiotics <2 weeks before the screening visit; had current active gastric or duodenal ulcer; had previous gastric malignancy; had alarm signs (e.g., abnormal weight loss, hematochezia, anemia, or significant bowel habit changes); had lactose intolerance; had uncontrolled comorbidity; were pregnant or breastfeeding; or were drug users or alcoholics. All enrolled patients received comprehensive information about this study, and informed consent was obtained before any study-related processes began. The study protocol was reviewed and approved by the institutional review board of our hospital (KHNMC IRB 2017-02-003).

### 2.2 Study design

This multicenter, prospective, randomized, double-blind, placebo-controlled clinical trial was conducted at Kyung Hee University Hospital and Vievis Namuh Hospital from April to December 2017. After screening of the inclusion and exclusion criteria, enrolled patients were assigned with consecutive allocation numbers, which were matched at a 1:1 ratio to a randomization code through a table of random numbers. Subjects were randomized into either the treatment group (fermented milk with *L paracasei* HP7 and *G glabra*) or placebo group (fermented milk only) once daily for 8 weeks. The status of *H pylori* infection was determined by ^13^C-UBT, CLO test, and histopathologic examination by gastric biopsy just before intake of study products and at 8 weeks after completion of administration. The participants, nurses, and researchers involved in this study were blinded to the interventions until the final database lock.

### 2.3 Study products and compliance

The study product was 1 bottle (150 mL) of fermented milk with or without probiotics and licorice extract. The study product contained 1.0 × 10^6^ CFU/mL *L paracasei* HP7 KCTC 13143BP as probiotics and 100 mg licorice extracted from deglycyrrhizinated roots and rhizomes of *G glabra* developed by Korea Yakult Co, Ltd (Seoul, South Korea). The placebo was prepared using the same ingredients, but without the *L paracasei HP7* KCTC 13143BP and *G glabra*, and had identical packaging to that of the study product in the treatment group. One bottle (150 mL) of the study product was taken once daily at the same time every morning. During the study period, patients recorded the time of study product intake and adverse events in daily diaries. All unused products had to be returned to the study site, and compliance was calculated at 4- and 8-week follow-up visits. Poor compliance was defined as taking an average of <75% of bottles.

### 2.4 Assessments and study endpoints

The primary endpoint was the gastric load of *H pylori* assessed on the 8th week. Gastric load of *H pylori* was quantified using ^13^C-UBT. ^13^C-UBT was performed after 4 hours of fasting using the UBT kit (Crystal Life Science, Bundang, South Korea). The 50-mg ^13^C-urea was dissolved in water and then administered orally. Baseline and 30-minutes breath samples were assayed with an infrared spectrometer that produced computer-generated results. Positive results were defined as a computer-generated δ^13^CO_2_ value ≥2%, and negative results as <2%.^[35]^ The secondary endpoints were histopathologic improvement assessed by the Sydney grading system, negative conversion of *H pylori* by the CLO test, and clinical improvement by 2 self-administered questionnaires.^[36–38]^ This Sydney grading system categorized gastritis according to intensity of neutrophil activity, chronic inflammation by mononuclear inflammatory cellular infiltrates, mucosal atrophy, intestinal metaplasia, and *H pylori* density (no = 0, mild = 1, moderate = 2, severe = 3).^[36,39]^ Four biopsy specimens were taken from both the lesser curvature (LC) and greater curvature (GC) sites of the antrum and corpus for histopathologic examination.^[40]^ Four scores at each site were summed up and ranged from 0 to 12 points. To assess clinical improvement, gastrointestinal symptom rating scale (GSRS) and World Health Organization Quality of Life Assessment Instrument (WHOQOL)-BREF questionnaires were used.^[37,38,41]^ The GSRS questionnaire includes 15 questions, which assess severity of gastrointestinal symptoms using a 4-point Likert scale, from 0 to 3, in 5 domains: indigestion, diarrhea, constipation, abdominal pain, and reflux.^[37]^ The symptom severity reported in the GSRS increases with increasing score. The WHOQOL-BREF has 26 items divided into 4 factor structures that include physical health, psychologic, social relationship, and environmental domains to measure a person's quality of life (QOL).^[38,41]^

### 2.5 Statistical analysis

The sample size was calculated assuming −2.91% quantitative change of ^13^C-UBT as the primary endpoint in the treatment group and −0.78% in the placebo group based on a previous study conducted under a similar setting.^[42]^ We estimated that a sample size of 56 subjects per group would have a statistical power of 80% and a 2-sided α-risk of 0.05. We planned to enroll 70 subjects in each group, assuming a 20% dropout rate. Efficacy was assessed by per protocol analysis and safety by intention-to-treat (ITT) analysis. The ITT analysis included all participants who had taken at least 1 dose of study drugs.

In comparing the demographic factors between the 2 groups, continuous variables were analyzed using Student *t*-tests and categorical variables using Chi-squared or Fisher exact tests. To assess the quantitative changes of primary and secondary endpoints before and after the study period in both groups, paired *t*-tests were performed. To evaluate values between the 2 groups at each time point, Student *t*-tests were used. All statistical tests were 2 sided, and a *P*-value <.05 was considered statistically significant. All statistical analyses were performed using the SAS/STAT software (SAS 9.4; SAS Institute, Cary, NC).

## 3 Results

### 3.1 Baseline characteristics

**Figure 1:**
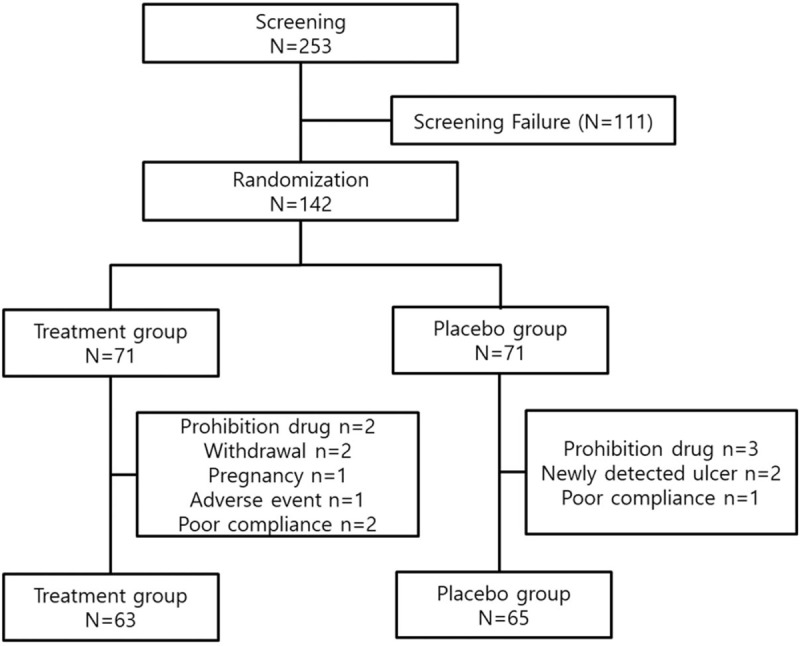
Patient flow diagram.

A total of 253 subjects were invited to participate in the study, and 111 subjects were ineligible as they did not meet the inclusion criteria. A total of 142 subjects consented and were enrolled in the study and then randomly allocated to the treatment group (n = 71) or placebo group (n = 71). After allocation, eight subjects in the treatment group were excluded because of prohibited medication use (n = 2), consent withdrawal (n = 2), newly confirmed pregnancy (n = 1), adverse event (n = 1), and poor compliance (n = 2). Six subjects in the placebo group were also excluded because of prohibited medication use (n = 3), newly detected gastric ulcer on gastroscopy (n = 2), and poor compliance (n = 1). Finally, 128 subjects (treatment group, n = 63; placebo group, n = 65) were analyzed (Fig. 1). For the baseline characteristics of the participants, age, sex, smoking, alcohol, occupation, and comorbidity were not different between the 2 groups (Table 1).

**Table 1:**
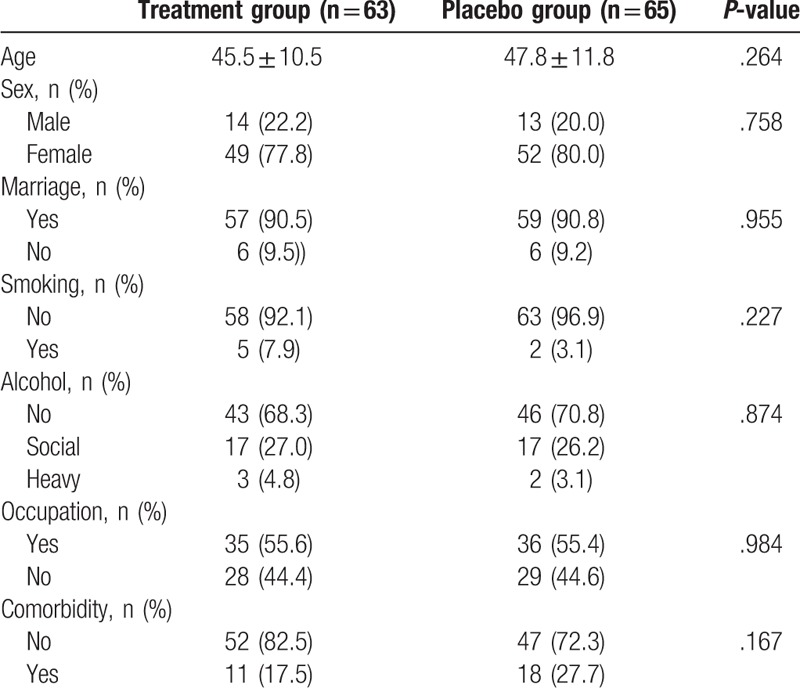
Baseline characteristics of the study subjects in the treatment and placebo groups.

### 3.2 Histologic efficacy analysis

Compared to baseline data, the quantitative value of ^13^C-UBT at 8 weeks was significantly reduced in the treatment group (from 20.8 ± 13.2% to 16.9 ± 10.8%, *P* = .035), but not in the placebo group (from 19.1 ± 12.7% to 16.9 ± 11.8%, *P* = .130) (Table 2). However, no significant difference was observed between the 2 groups at baseline and 8 weeks. Two patients in the treatment group and 1 in the placebo group converted to negative status of *H pylori* infection measured by ^13^C-UBT after 8 weeks of administration, which was not significant (*P* = .616).

**Table 2:**
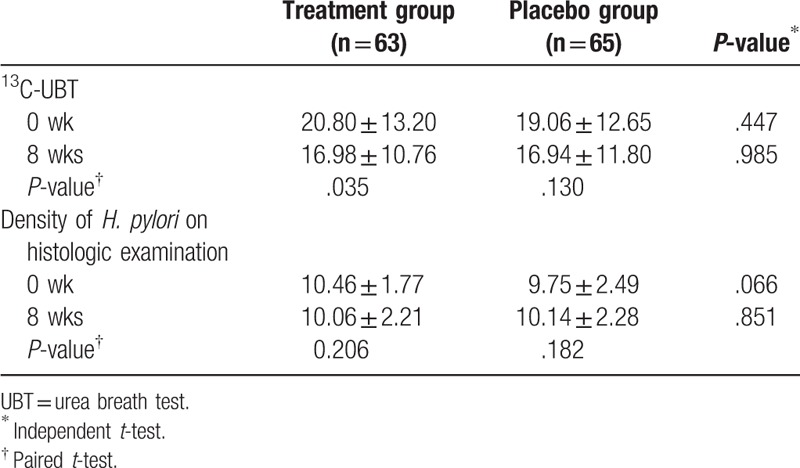
Efficacy analysis for viral load of the *Helicobacter pylori* infection before (0 week) and after (8 weeks) the treatment.

**Figure 2:**
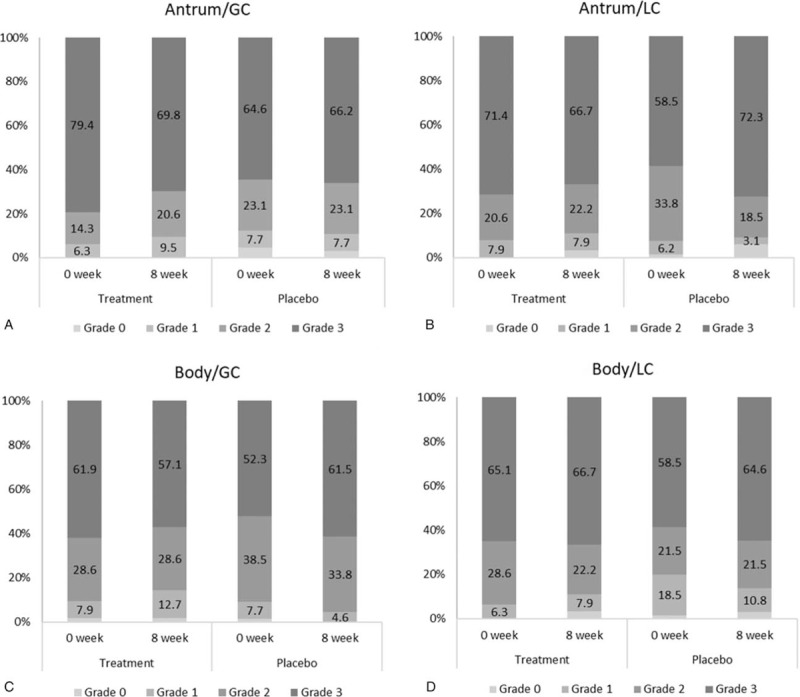
The proportion (%) of *Helicobacter pylori* density divided by the Sydney classification on histopathologic examination at the (A) antrum/greater curvature (GC), (B) antrum/lesser curvature (LC), (C) body/greater curvature, and (D) body/lesser curvature before (0 week) and after (8 weeks) the treatment.

After 8 weeks of intervention, *H pylori* density on histologic examination showed no significant difference within and between groups in the total score of the Sydney classification (Table 2). In the subgroup analysis, severe inflammation (grade 3) decreased in the treatment group at the antrum/GC, antrum/LC, and body/GC (Fig. 2). However, severe inflammation (grade 3) rather increased in the placebo group at the antrum/GC, antrum/LC, body/GC, and body/LC (Fig. 2). In chronic inflammation judged from infiltration of inflammatory cell, the degree of inflammation improved significantly in the treatment group (*P* = .013), but not in the placebo group. The neutrophil activity deteriorated significantly in the placebo group (*P* = .003), but not in the treatment group. There was no significant alteration in mucosal atrophy and intestinal metaplasia in both groups (Table 3).

**Table 3:**
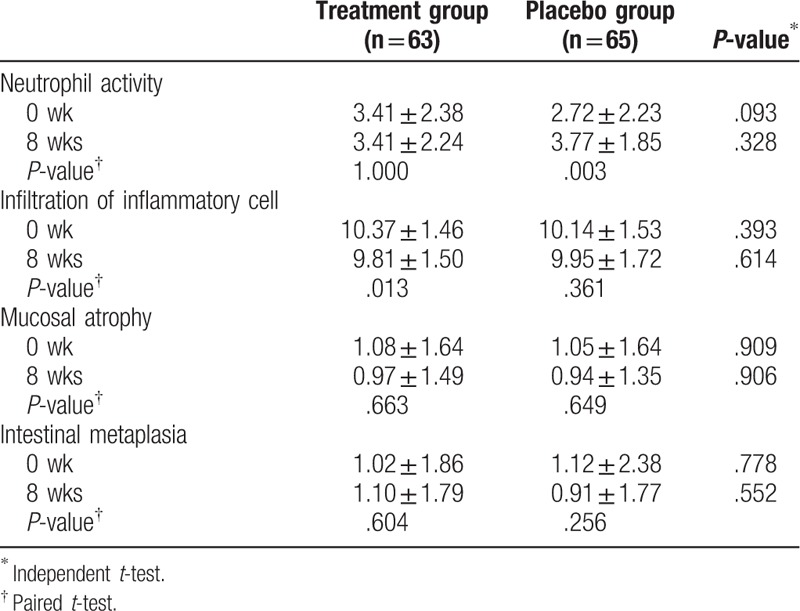
Histopathologic analysis of *Helicobacter pylori* infection before (0 week) and after (8 weeks) the treatment.

### 3.3 Clinical efficacy analysis

Overall gastrointestinal symptoms measured by GSRS improved significantly in the treatment group (from 2.6 ± 3.9 to 1.9 ± 2.6, *P* = .049), but not in the placebo group (from 3.2 ± 3.4 to 2.4 ± 2.8, *P* = .106) (Table 4). In the WHOQOL-BREF, the QOL score was significantly better in the physical health domain of the treatment group (*P* = .029); however, the QOL scores were not significantly improved in the psychologic, social relationship, and environmental domains. There was no significant improvement in all four domains of the placebo group. However, between the treatment and placebo groups over the study period, no significant differences were found for clinical symptoms measured by the GSRS and WHOQOL-BREF.

**Table 4:**
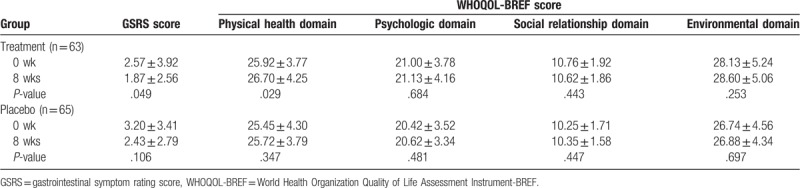
Clinical efficacy analysis before (0 week) and after (8 weeks) the treatment.

### 3.4 Safety and tolerability

Adverse events developed in five patients (7.0%) in the treatment group (n = 71) and in 6 (8.5%) in the placebo group (n = 71) without significant difference (*P* = .771) (Table 5). Common adverse events were minor infection/inflammation (5.6%), gastrointestinal disorders (4.2%), dermatologic disorders (2.8%), and others (2.8%), without significant difference between the 2 groups. All adverse events were mild in intensity without serious events, but one patient in the treatment group abandoned this trial because of allergic contact dermatitis, which has no direct association with this trial. The mean compliance rate was 98.2 ± 3.2% in the treatment group and 97.8 ± 4.5% in the placebo group (*P* = .509). Two patients in the treatment group and 1 patient in the placebo group showed poor compliance, defined as <75% intake rate (*P* = .509).

**Table 5:**
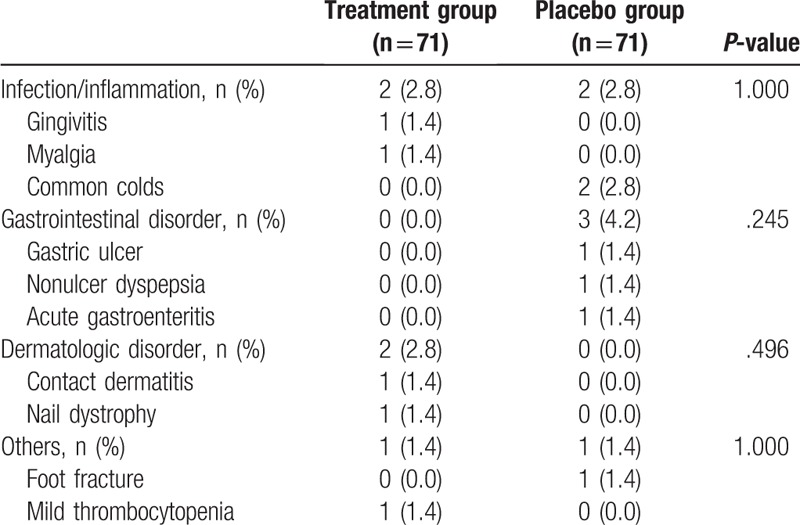
Adverse events reported over the entire treatment period through intention-to-treat analysis.

## 4 Discussions

The effects of probiotic supplementation along with the standard triple regimen have been evaluated in the management of *H pylori*.^[43]^ Although several studies have reported the anti-*H pylori* activity of *Lactobacillus* alone in vitro or in experimental models, few human studies have evaluated their effects.^[44–47]^ In the present study, the beneficial effect of fermented milk containing *L paracasei* HP7 and herbal extract (*G glabra*) for 8 weeks was evaluated as single agent in patients with *H pylori* infection. We showed reduction in *H pylori* urease activity and gastric inflammation only in the treatment group. In our study, severe inflammation significantly decreased and the degree of chronic inflammation significantly ameliorated only in the treatment group. Higher density of *H pylori* in the gastric mucosa is related to more severe gastritis and increased incidence of peptic ulcers; therefore, reduction of the density may suppress the development of pathologic conditions in the gastric mucosa.^[48]^ Furthermore, our combination regimen also led to clinical improvement in gastrointestinal symptoms and QOL. Therefore, our study product may have a beneficial effect in patients with *H pylori* infection, as it reduced *H pylori* density detected by the quantitative assessment and improved histologic inflammation, even though it failed to eradicate *H pylori*.

The main pathogenesis of inflammation induced by *H pylori* is characterized by neutrophil infiltration into the epithelial cell layer.^[49]^ Interleukin (IL)-8 is known as a key modulator, which initially leads to the migration and activation of neutrophils in *H pylori*-infected gastric epithelium.^[50]^ After IL-8 expression, local release of proinflammatory cytokines and chemokines also induced recruitment of monocytes and lymphocytes and increased *H pylori* attachment to the surface of the gastric epithelium.^[50,51]^ Persistent inflammatory process caused by chronic *H pylori* infection might bring about mucosal damage, such as atrophic change, intestinal metaplasia, ulcer, and even cancer.

There is no golden standard in measuring *H pylori* load; however, ^13^C-UBT has been used as a quantitative assessment of *H pylori* density. In previous studies, *H pylori* load was measured indirectly by the UBT, because the bacterial urease activity is correlated with values of UBT.^[52,53]^ Therefore, in our study, a decrease in UBT values in the treatment group could reflect a decrease in the *H pylori* bacterial load. *H pylori* load may be clinically important, as Tokunaga et al reported that the higher the increase in *H pylori* density, the greater the risk of *H pylori*-associated disease.^[54]^ In contrast, we failed to show significant change in *H pylori* load measured by histopathologic examination. However, unequal distribution of *H pylori* in the gastric mucosa might make histopathologic examination less reliable than UBT in the measurement of *H pylori* density.^[55]^

The role of probiotics in the treatment of *H pylori* infection has been more widely known as a supplement to standard regimens than as a main therapy.^[10,11,56,57]^ Currently, there are several clinical trials on probiotics alone to identify their anti-*H pylori* effect, as it may have beneficial effects on gastric *H pylori* through several possible mechanisms.^[10,11]^ Probiotics may prevent colonization of *H pylori* by competing with *H pylori* for adhesion to the epithelium, strengthen gastric mucosal barrier by synthesizing antimicrobial compound, and stimulate mucin production.^[58–60]^ Probiotics also reduce gastric activity and regulate the balance between proinflammatory and antiinflammatory cytokines.^[13,61]^ Most effective strain of probiotics has not been determined yet for the management of *H pylori* infection, but *Lactobacillus* species showed proven efficacy because they are resistant to acidic environment, pancreatic enzyme, and bile salts and release lactic acid inhibiting the adhesion of *H pylori* to the cells.^[43,62–64]^ In our study, *L paracasei* was used because it can produce a remarkable amount of lactic acid, which is regarded as the origin of anti-*H pylori* activity,^[65]^ and has better immunomodulatory function in preventing intestinal inflammation than *L plantarum* or *L rhamnosus*.^[16,66]^ Furthermore, *L paracasei* inhibited the elevation of IL-8 and regulated upon activation, normal T-cell expressed and presumably secreted (RANTES) released from the *H pylori*-infected gastric epithelium.^[62,67]^

In our study, severe inflammation (grade 3) improved dominantly in the antrum than in the corpus of the stomach after the 8-week treatment, which was consistent with previous findings where probiotics improved *H pylori*-induced histopathologic features predominantly in the antrum.^[68]^ The gastric antrum is a major colonization site of *H pylori* as there are few acid-secretory parietal cells^[48]^; therefore, probiotics may have more beneficial effects in *H pylori* infection in the antrum than the body. The gut microbiome may be altered by a standard regimen for *H pylori* eradication including high-dose PPI and 2 antibiotics.^[10]^ Previous meta-analysis about the role of probiotics in the *H pylori* eradication demonstrated that overall adverse effect rates significantly decreased with probiotics combination by stabilizing or restoring gut microflora.^[6]^ In our study, general gastrointestinal symptoms measured by GSRS and QOL measured by WHOQOL-BREF improved significantly in the treatment group compared with those in the placebo group, which may be explained by the beneficial effect of probiotics on gut microbiota.

*Glycyrrhiza glabra*, commonly known as licorice, has been traditionally used to treat patients with peptic ulcers in Oriental medicine^[27]^ and showed anti-*H pylori* and antiulcer activities in in vitro studies.^[30,33,34,69]^ Aqueous extract from the roots of this plant was also reported to suppress *H pylori* activity through antiadhesion effects of *H pylori* to the gastric epithelium and antioxidative effects against gastric mucosal injury.^[31,33,69]^ These results recently led to produce a commercial compound, namely GutGard, a flavonoid-rich root extract of *G glabra*.^[42,69,70]^ Puram et al reported a 48% (24/50 subjects) *H pylori* eradication rate in patients taking 150 mg GutGard alone for 60 days compared with only 2% (1/50 subjects) *H pylori* eradication rate in the placebo group measured by ^13^C-UBT.^[42]^ In our study, no eradication occurred in both treatment and placebo groups; however, *H pylori* bacterial load was decreased in the treatment group only. Further study is necessary to validate our findings as we used less dose of *G glabra* than that in the GutGard study.^[42]^

There were several limitations in this study. First, we could not evaluate the individual effect of probiotics or licorice because we did not perform a clinical trial to evaluate each of its efficacy. Second, we found only significant interval change in the intragroup analysis, but not in the intergroup analysis. This discrepancy may be explained by insufficient duration of therapy or the relatively small dosage. Previous studies reported that efficacy of probiotics could vary according to the duration of therapy; however, the optimal duration of therapy is still uncertain.^[71,72]^ Third, we did not assess intestinal microbiome analysis results after administration of the study product. If fecal microbiome analysis is conducted, more information on altered composition by supplying *L paracasei* and *G glabra* can be gained. Lastly, we did not evaluate the effect of this combination as an adjunctive agent on improving *H pylori* eradication rates and ameliorating adverse events associated with the standard regimen. Furthermore, the role of this mixture in the prevention of clinical consequences related to *H pylori* infection requires further evaluation.

In conclusion, the combination of fermented milk containing *L paracasei* and *G glabra* reduced *H pylori* density and improved histologic inflammation. However, their mechanisms of action should be elucidated, and their role in the prevention of clinical consequences related to *H pylori* infection requires further evaluation.

## Author contributions

**Conceptualization:** Jae Myung Cha.

**Data curation:** Jin Young Yoon, Hyung Kyung Kim.

**Formal analysis:** Jin Young Yoon.

**Investigation:** Hyung Kyung Kim, Min Seob Kwak.

**Methodology:** Jung Won Jeon.

**Resources:** Min Seob Kwak, Jung Won Jeon, Hyun Phil Shin.

**Supervision:** Jae Myung Cha, Seong Soo Hong.

**Writing – original draft:** Jin Young Yoon.

**Writing – review & editing:** Jae Myung Cha.
